# Discovering functional modules by identifying recurrent and mutually exclusive mutational patterns in tumors

**DOI:** 10.1186/1755-8794-4-34

**Published:** 2011-04-14

**Authors:** Christopher A Miller, Stephen H Settle, Erik P Sulman, Kenneth D Aldape, Aleksandar Milosavljevic

**Affiliations:** 1 Graduate Program in Structural and Computational Biology and Molecular Biophysics, Baylor College of Medicine, Houston, Texas, USA; 2 Department of Molecular and Cellular Biology, Baylor College of Medicine, Houston, Texas, USA; and Department of Radiation Oncology, the University of Texas M. D. Anderson Cancer Center, Houston, Texas, USA; 3 Department of Radiation Oncology, the University of Texas M. D. Anderson Cancer Center, Houston, Texas, USA; 4 Department of Pathology, the University of Texas M. D. Anderson Cancer Center, Houston, Texas, USA; 5 Graduate Program in Structural and Computational Biology and Molecular Biophysics; and Department of Molecular and Human Genetics, Baylor College of Medicine, Houston, Texas, USA

## Abstract

**Background:**

Assays of multiple tumor samples frequently reveal recurrent genomic aberrations, including point mutations and copy-number alterations, that affect individual genes. Analyses that extend beyond single genes are often restricted to examining pathways, interactions and functional modules that are already known.

**Methods:**

We present a method that identifies functional modules without any information other than patterns of recurrent and mutually exclusive aberrations (RME patterns) that arise due to positive selection for key cancer phenotypes. Our algorithm efficiently constructs and searches networks of potential interactions and identifies significant modules (RME modules) by using the algorithmic significance test.

**Results:**

We apply the method to the TCGA collection of 145 glioblastoma samples, resulting in extension of known pathways and discovery of new functional modules. The method predicts a role for *EP300 *that was previously unknown in glioblastoma. We demonstrate the clinical relevance of these results by validating that expression of *EP300 *is prognostic, predicting survival independent of age at diagnosis and tumor grade.

**Conclusions:**

We have developed a sensitive, simple, and fast method for automatically detecting functional modules in tumors based solely on patterns of recurrent genomic aberration. Due to its ability to analyze very large amounts of diverse data, we expect it to be increasingly useful when applied to the many tumor panels scheduled to be assayed in the near future.

## Background

Tumor characterization projects are beginning to produce a large volume of data about genomic, epigenomic, and gene expression aberrations in tumor samples. This unprecedented volume of information has the potential to transform our understanding of cancer biology, reveal new biomarkers and drug targets, and accelerate the development of new cancer therapies. One recent genome-wide tumor characterization effort revealed recurrent somatic aberrations in 91 glioblastoma (GBM) tumors [1]. In that study, mapping of recurrent aberrations in individual genes to known pathways was used to link three core pathways to cancer progression.

A key question is how to extend integrative analysis of somatic genomic aberration data to expand known cancer pathways and interactions, or discover completely new modules (sets of related genes). Such inference has been done extensively using gene expression arrays, both in yeast and humans [2-4], but due to the dynamic nature of the transcriptome it is often difficult to separate causative events from their effects [5,6]. Instead, we develop an algorithm that infers functional modules directly from mutational patterns. This approach resembles, in some aspects, the mapping of epistatic or synthetic lethal genetic interactions in yeast [7].

Specifically, we focus on patterns of recurrent and mutually exclusive aberrations (RME patterns). Previous analyses of large tumor panels have discovered that alteration of genes comprising a specific functional module are often observed across a sample collection, but are almost never concurrently found in the same tumor. Examples of these modules include *EGFR *and *KRAS *in lung adenocarcinomas [8], the recurrent fusion of *TMPRSS2 *and various *ETS *oncogenes in prostate cancer [9], and *TP53 *and *MDM2 *in many different types of malignancies [10]. One explanation for these patterns is that there are functional relationships between the genes. Specifically, an aberration in one of the genes may result in the development of a key tumorigenic phenotype, removing the selective pressure for mutation of the others.

The key insight is that these RME patterns may be used to identify groups of genes that are functionally related. This concept was explored in 2008 in the context of cancer by Yeang et al., who utilized data from the Catalogue of Somatic Mutations in Cancer (COSMIC) to identify functional relations among mutated genes [11]. Their attempt was hampered by the extremely small number of genes assayed and restriction to only examining point mutations. Their method of establishing the significance of a detected pattern was also limited by the need for computationally expensive permutation testing that does not scale to the large networks that are being produced from next-generation assays.

**Figure 1:**
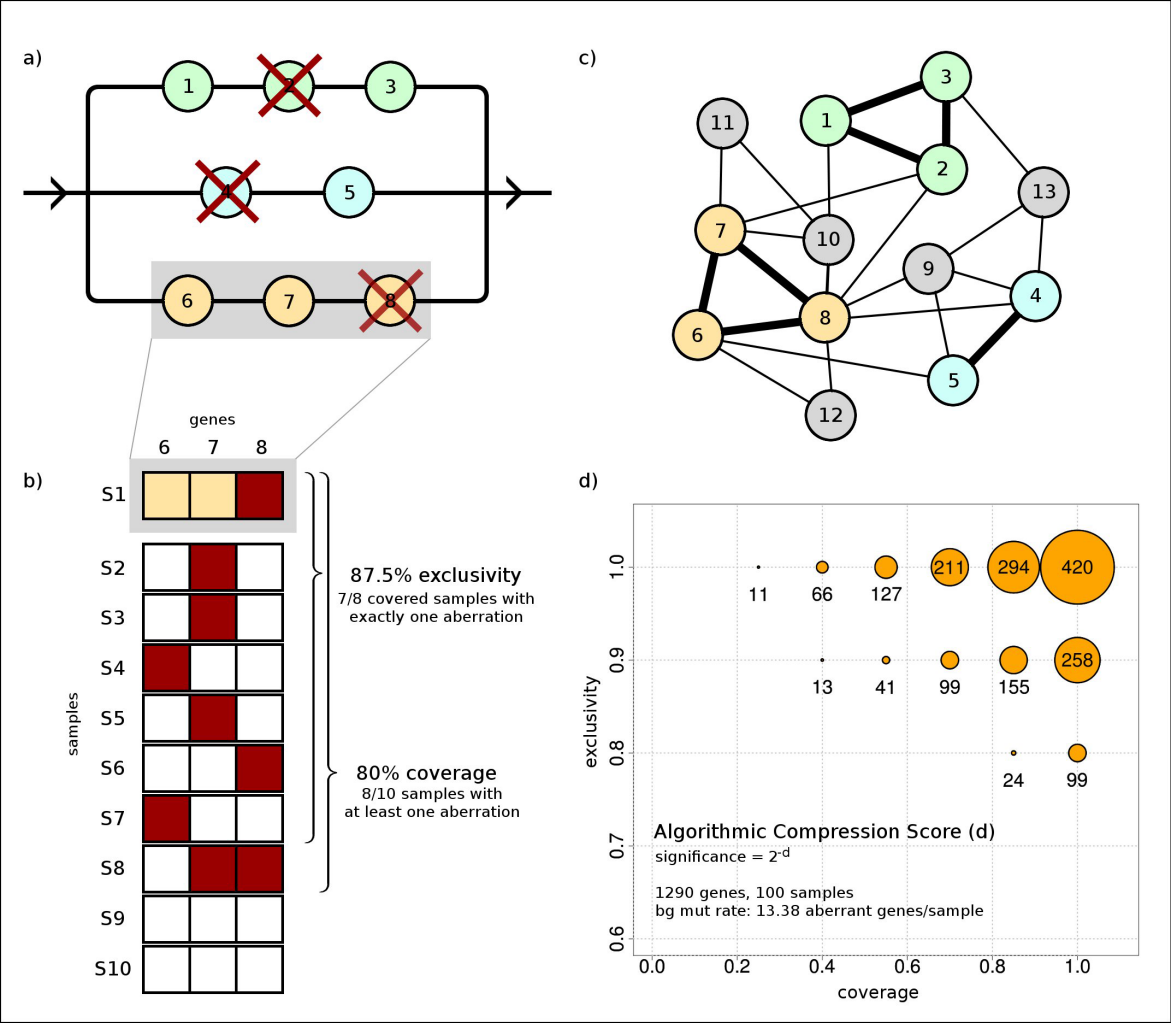
**Overview of RME Module Detection**. **a) **An example of a structural reliability model of progression of a particular tumor type. Cancer progression in this example requires aberrations in each of the three distinct functional modules (three horizontal lines). If mutated genes (crossed out in red) occur in all three modules, the connection between the left and right part of the structural model will be lost, indicating failure (cancer). **b) **A module may be disrupted by different aberrations in distinct tumor samples. One measure of an RME pattern is coverage, defined as the percentage of samples that contain at least one aberration within the module. Another measure of the pattern is exclusivity, defined as the percentage of covered samples that contain exactly one aberration within the module. An aberration in one of the genes within a specific RME module removes selective pressure of aberrations in other genes within the same module, giving rise to the exclusivity. **c) **Example network where nodes represent genes and edge thickness represents the level of exclusivity. The search for RME patterns starts by constructing such a graph using the Winnow algorithm. This graph indicates three potential RME modules. The node colors and numbers correspond to those in panel **a**. **d) **The significance score for RME patterns is dependent on both exclusivity (y-axis) and coverage (x-axis). Shown is the RME algorithmic compression score, d, for a three-gene RME module across 100 samples with aberrations equally distributed, assuming background frequency of 13.38 aberrant genes per sample (see section 2.3 andAdditional file 1). According to the algorithmic significance test, the significance of an RME pattern is 2^-d^.

To address these issues, we developed a new method for detecting RME patterns, which we formalized by using structural reliability models [12]. Specifically, as illustrated in Figure 1a/b, the RME patterns correspond to modules of the "OR" type in these models, where abrogating the function of one member in each module is sufficient for failure. In our case, "failure" refers to the development of a tumor phenotype. We hypothesized that these RME patterns are sufficiently informative to enable the discovery of cancer-related functional modules without using any prior information. We then tested this hypothesis by designing an algorithm for accurate and computationally efficient detection of these modules. Our tool uses the Winnow algorithm for network construction and establishes significance via the algorithmic significance method, eliminating the need for costly permutation testing. Through simulation experiments, we show that this algorithm scales to very large data sets and evaluate the types of modules that are discoverable using data that will be generated by large tumor characterization projects. We validate our method by applying it to a data set currently available though the TCGA data portal, which consists of mutation and copy number data collected from a cohort of 145 primary GBM tumors. The algorithm identifies known modules from core GBM pathways, extends these modules with new members, and discovers new modules that may inform future studies.

## Methods

### Creating a mutation matrix

**Figure 2:**
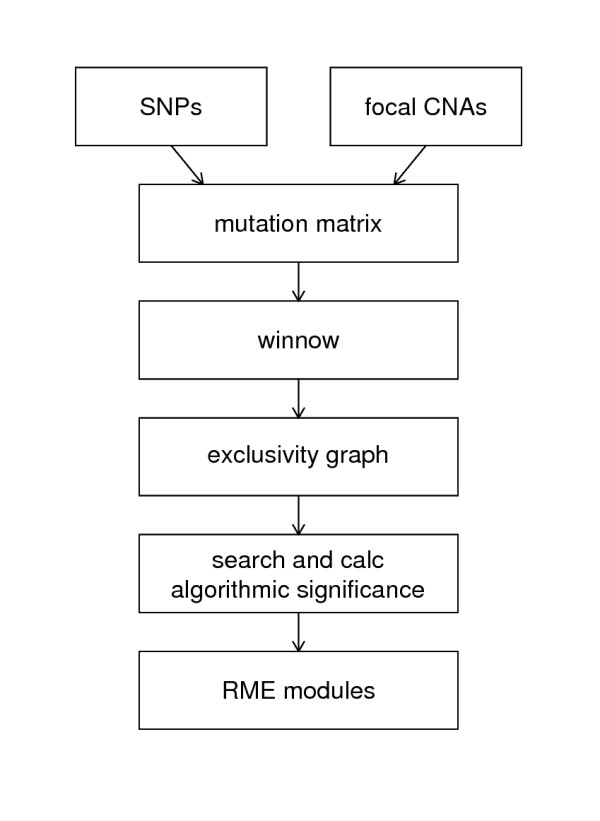
**Analysis Pipeline**. In a preprocessing step, validated SNPs and focal CNAs are combined into a mutation matrix. This matrix is fed into the winnow algorithm, which scores each gene pair by exclusivity, indicated by edge scores in a graph. This graph is then searched for modules up to a specified size and the algorithmic significance is calculated for each potential module. Finally, the most significant modules are reported.

We designed our algorithm to be capable of utilizing many disparate sources of mutational data, including single-nucleotide polymorphisms, copy-number alterations, and epigenomic modifications. In a pre-processing step, these diverse data types were converted into a single two-dimensional binary "mutation" matrix (Figure 2).

Data was obtained from the The Cancer Genome Atlas Data Portal (http://tcga-data.nci.nih.gov/). A complete listing of samples used can be found in Table 1 in Additional file 1. Point mutations identified by resequencing were filtered such that only non-synonymous, validated mutations remained. Genes driving copy number alterations were detected using normalized probe-level data from Agilent 244A copy number arrays. These were processed to infer regions of amplification and deletion using circular binary segmentation as implemented in the R package DNAcopy [13]. Log-ratio thresholds for amplification and deletion were set at 1.5 standard deviations from the mean probe intensity. These were intersected with peaks of recurrent copy-number change identified by the RAE algorithm [14], then copy number variants were removed and driver genes were selected as described in Additional file 1.

These two forms of data were then merged into a two-dimensional mutation matrix. Each gene in each sample was checked against these single nucleotide and copy number mutations and a matrix was created such that if sample *i *contained an alteration in gene *j*, the position *x*_*i,j *_in the matrix was equal to 1, otherwise it was set to 0. This matrix is available at http://brl.bcm.tmc.edu/rme/gbm.dat

### Constructing a gene network with Winnow

The first step in our module detection pipeline was to filter the mutation matrix and retain only genes that meet a set frequency of recurrence, as genes altered in only one or a few samples do not contain enough information to calculate meaningful exclusivity scores.

A possible next step would be to calculate the exclusivity score between each pair of genes, defined as the number of samples where exactly one of the pair is mutated divided by the number of samples where at least one of the pair is mutated. (Figure 1b). These data could be used to create a network where each node is a gene and each edge weight is the exclusivity between the genes. The highly connected sub-networks would then be used as a starting point for a focused combinatorial search for modules. The disadvantage of this approach is that the networks quickly becomes much too large and densely connected to effectively identify sub-networks.

Thus, we used an online-learning linear threshold algorithm called Winnow to detect signals of exclusivity against the noisy background of passenger mutations in many irrelevant genes [15]. Its speed and insensitivity to irrelevant attributes allowed us to aggressively filter the output scores before generating a network, resulting in smaller, higher quality networks than pairwise exclusivity.

The Winnow algorithm was run in an online setting, using one gene as a classifier and the rest of the mutation array as training data. In the first winnow run, all the bits in the array were flipped, such that we calculated how well each aberration in the classifier is predictive of non-aberration in each gene of the matrix. Then, we flipped the bits of the classifier, such that we calculated how well each non-aberration in the classifier was predictive of aberration in each gene of the matrix. The resulting weights were used to score the edges of the graph, then low-scoring edges were removed.

Since the range of weights for each run was determined by how quickly Winnow finds an optimal classifier, we did not use an absolute threshold value when removing edges. Instead, for each classifier gene, we took the second highest weight and retained all edges with a score greater than or equal to that value.

### Identifying candidate modules

We then used each gene in the network as a starting point in a greedy local combinatorial search for RME modules, such that we evaluated all possible connected modules with size below the specified limit. We report those that have algorithmic significance above a predetermined threshold, based on the size of the input data (Figure 1C). These potential RME modules are binned by number of genes and sorted by significance value. The module with the largest size and highest significance (as described in the next section) is kept, and all other modules containing any of the same genes are discarded. This process is repeated until all bins are empty.

### Evaluating modules by performing an algorithmic significance test

The problem of determining whether a module (subset of genes) contains a significant RME pattern of aberrations can be addressed using probabilistic models or heuristic scores. Both approaches would generally require establishment of extremely low significance values (pre-Bonferroni correction), which would in turn require many cycles of computationally demanding permutation testing. To eliminate this bottleneck, we employ a new implementation of the computationally much less demanding algorithmic significance test [16], which has recently been applied in the context of Hidden Markov Models [17-19], but is also applicable as a general method for pattern discovery [20,21]. As illustrated in Figure 1d, the algorithm determines significance values directly.

Let *k *be the number of samples, *m *be the number of genes in a module, and let X be a *k *times *m *matrix of binary values, with each value indicating presence (*x*_*i,j *_= 1) or absence (*x*_*i,j *_= 0) of an aberration of the *j*-th gene in the *i*-th sample. The algorithmic significance test compares the number of bits required to encode the binary matrix X by the RME Algorithm to the number of bits required to encode the matrix under the null hypothesis. The RME algorithm attempts to encode the data in fewer bits by using the assumption that mutations occur at an unusually high frequency in a mutually exclusive fashion, vs. the Null Algorithm (corresponding to the null hypothesis) which assumes that aberrations occur independently at their background frequencies.

The presence of an RME pattern (Figure 1b) will allow the RME algorithm to encode the matrix X significantly more concisely. To minimize overall encoding length, the RME Algorithm is provided the identity of the *m *genes out of the total of *n *genes assayed (encoded in *m *log(n) bits, an implicit penalty that corrects for multiple testing), and the counts of aberrations for each sample, *a*_*i*__,0_, *i *= 1,...,*k *and counts of aberrations for each gene, *b *_0,__*j*_,j = 1,...,*m *(encoded in *k *log*(*m*) and *m *log*(*k*) bits respectively, where log* denotes iterated logarithm). Using this information, the RME Algorithm first sorts the samples, then the genes by their counts of aberrations, placing the most frequently altered samples at the top of the matrix, and the genes with most aberrations at the beginning of each row. As a penalty for this sorting, we reduce the score by the number of bits necessary to represent the new sorted order.

The algorithm then examines the sorted matrix row by row in a left to right order, keeping track of how many aberrations have been observed, and calculates a probability of observing an aberration in the next cell of the matrix and encoding the bit optimally according to the calculated probability. To describe how the probability is calculated, we first introduce additional notation. Let *p*(*x*_*i,j *_= 1) denote the number of unobserved mutations divided by the number of unobserved positions remaining in the matrix. Let *a*_*i,j *_and *b*_*i,j *_denote the number of unobserved aberrations in the current gene and sample respectively.

Then, we can encode elements of X according to the following probability distribution: If *a*_*i,j *_and *b*_*i,j *_are both larger than 0, and a one has not been observed in this row yet, we use the following formula (derived by applying Bayes' rule):

else we estimate that the probability is very low (but not equal to zero in order to avoid infinitely large penalties):

In contrast, the Null algorithm encodes optimally assuming that the *k *genes contain aberrations at background frequency (no enrichment), denoted *p*_*NULL *_(1), and that mutations occur independently in each of the k genes.

The encoding length difference between the null and RME algorithms and the algorithmic significance are calculated in the following two steps:

**Step 1**. Encode the binary aberration matrix.

Set *d' *to 0. Examine the aberration matrix row by row, in left to right order incrementing *d' *as follows in each cell (*i,j*):then

If *x*_*i,j *_= 1 then:

Else,

where log denotes binary logarithm.

**Step 2**. Account for additional information (including implicit correction for multiple testing) and calculate significance.

Calculate significance value 2^-*d*^.

### Whole-genome simulations

In order to benchmark the performance of this algorithm, we ran simulations on synthetic data sets. When generating sets with the same size as the current glioblastoma data (145 samples, 1290 genes), the actual distribution of mutations from the TCGA data was used to create random matrices. We simulated larger data sets using the knowledge that the current gene list is heavily biased towards known and frequently-altered oncogenes, so we compensated by assuming that 0.1% of newly considered genes will have a mutation frequency greater than 0.2, 0.9% will have frequency between 0.2 and 0.1, and 99% will have frequency less than 0.1.

We then used a binning procedure, where we started with the empirical GBM distribution, and calculated the proportion of mutations in each bin. To compensate for the fact that the distribution is heavily biased towards low-frequency mutations, we used bins of size 1% until we reached the tenth percentile, then used bins of size 5% to allow for some variability. We then distributed the specified proportion of aberrations randomly within each bin. We tested coverage levels between 50 and 100%, and generated RME Modules such that the number of alterations matched the given coverage level, exclusivity was 100%, and each gene was altered in a random number of samples that exceeded the minimum threshold.

### Determination of prognostic significance

Affymetrix HG133-based GeneChip mRNA expression profiling data from two published datasets, the TCGA ("TCGA", n = 260) and the Erasmus Medical Center, Netherlands ("Erasmus", n = 153) were obtained as raw intensity files (.CEL files) and normalized [1,22]. Samples were included for all cases in which clinical data were available (patient age at diagnosis, tumor grade, survival time, and vital status) and for which the diagnosis was primary glioblastoma. Mapping of Affymetrix GeneChip probes was performed using custom chip definition files (CDF) based on the NCBI Entrez Gene v.11 (http://brainarray.mbni.med.umich.edu/Brainarray/. Database/CustomCDF/genomic_curated_CDF.asp) and probesets were summarized by median intensity [23]. Recursive partitioning analysis was performed to separate samples as either high or low expression. Univariate comparison of survival by *EP300 *expression was performed by the Kaplan-Meier method [24] with significance determined using the log-rank test. Multivariate analysis was performed using the Cox proportional hazards model [25]. All analyses were conducted using JMP Genomics 4.0.

### Implementation and availability

Implementation of the algorithm was done using Ruby and Bash. The core algorithm is available for download at http://brl.bcm.tmc.edu/rme/ or for use through the Genboree/Galaxy portal at http://www.genboree.org/galaxy, under Tools > Pattern Discovery. Documentation and example files are included at each location.

## Results

### Discoverability of RME modules using current and anticipated TCGA project data

To determine how well our method detects RME modules over the background noise of passenger mutations, we ran experiments on synthetic data using several different parameter sets. As described in the methods, we created a randomized mutation matrix then added an RME module consisting of two to five genes. One thousand simulations were run for each parameter set to determine whether the seeded RME module could be detected. We measured sensitivity by the fraction of simulations where the seeded module was detected above the significance threshold. We measured precision by the fraction of simulations where the algorithm detected the seeded genes as more significant than any other module.

**Figure 3:**
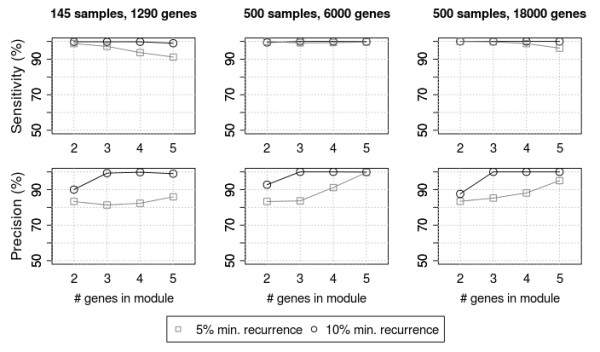
**Simulation Results**. One thousand simulations were run using varied numbers of genes and samples, for 5% and 10% recurrence thresholds. As sample size and the number of genes assayed increase, our algorithm retains the ability to detect RME modules with high sensitivity and precision.

Genes altered in only a few samples did not contain enough information to calculate meaningful exclusivity scores, so we tested two different recurrence thresholds. When considering only genes that are altered in at least 10% of the samples, the algorithm had high sensitivity and precision, with smaller modules being more susceptible to false positives that arise by chance (Figure 3, left column)

We then evaluated the characteristics of pathways that are discoverable using the data that is to be generated by future stages of the TCGA project. We increased the number of samples to 500 and increased the number of resequenced genes to either the 6000 currently being evaluated in TCGA Phase 2, or the ~18000 that may be examined with whole-exome coverage (Figure 3, center and right panels). The 6000-gene tests showed that both metrics increase, allowing us to accurately detect modules even at lower recurrence rates. When testing 18000 gene assays, we randomly generated more gene pairs with good exclusivity, which had a slight negative impact on sensitivity at lower recurrence levels, but overall, the method continued to perform well.

### Comparison to other methods

We also compared the performance of our algorithm to previously published methods based on calculating p-values for exclusivity from a hypergeometric test or from a log-likelihood ratio [11]. We benchmarked the performance of all three methods on a synthetic data set of 145 samples and 1290 genes, with two-gene modules seeded in. These were created as described in the Methods section. We find that all three methods have high sensitivity, but the two comparison methods have very low precision, and are prone to reporting false positives (Figure 2 in Additional file 1).

This can be explained as follows: The formula for calculating the likelihood ratio between the frequency of joint mutations relative to the best simpler model, as given in Yeang, is:

where the denominator is the empirical frequency of mutations in the first and second genes respectively, and the numerator is the empirical frequency of co-mutation. Thus, for two genes that are not mutated in the same samples, the likelihood ratio is 0 whether they each have one mutation, or they both have many mutations. Because of this characteristic, the likelihood method almost always reports false positive modules of genes that are exclusive by chance. Such modules usually have much lower coverage than the true seeded module. Since our algorithmic significance test considers recurrence as well as mutual exclusivity, it much more reliably excludes these false positives. The hyper-geometric p-value calculations described in Yeang suffer from a similar problem.

These other methods are also orders of magnitude slower than algorithmic significance, since they require many rounds of permutation testing to do multiple testing correction. Averaged across ten trials, the likelihood-based method had an average runtime of 899.377s, the hypergeometric method had an average runtime of 409.543s, and the algorithmic significance method had an average runtime of 0.779s. As described in the Methods section, algorithmic significance handles the problem of multiple testing using a penalty that takes very little time to compute. In contrast, both of the other methods require a step where the input data is permuted 1000 times and the number of combinatorial patterns is assessed. Unsurprisingly, this step makes these methods much slower.

### Application to glioblastoma tumors

**Figure 4:**
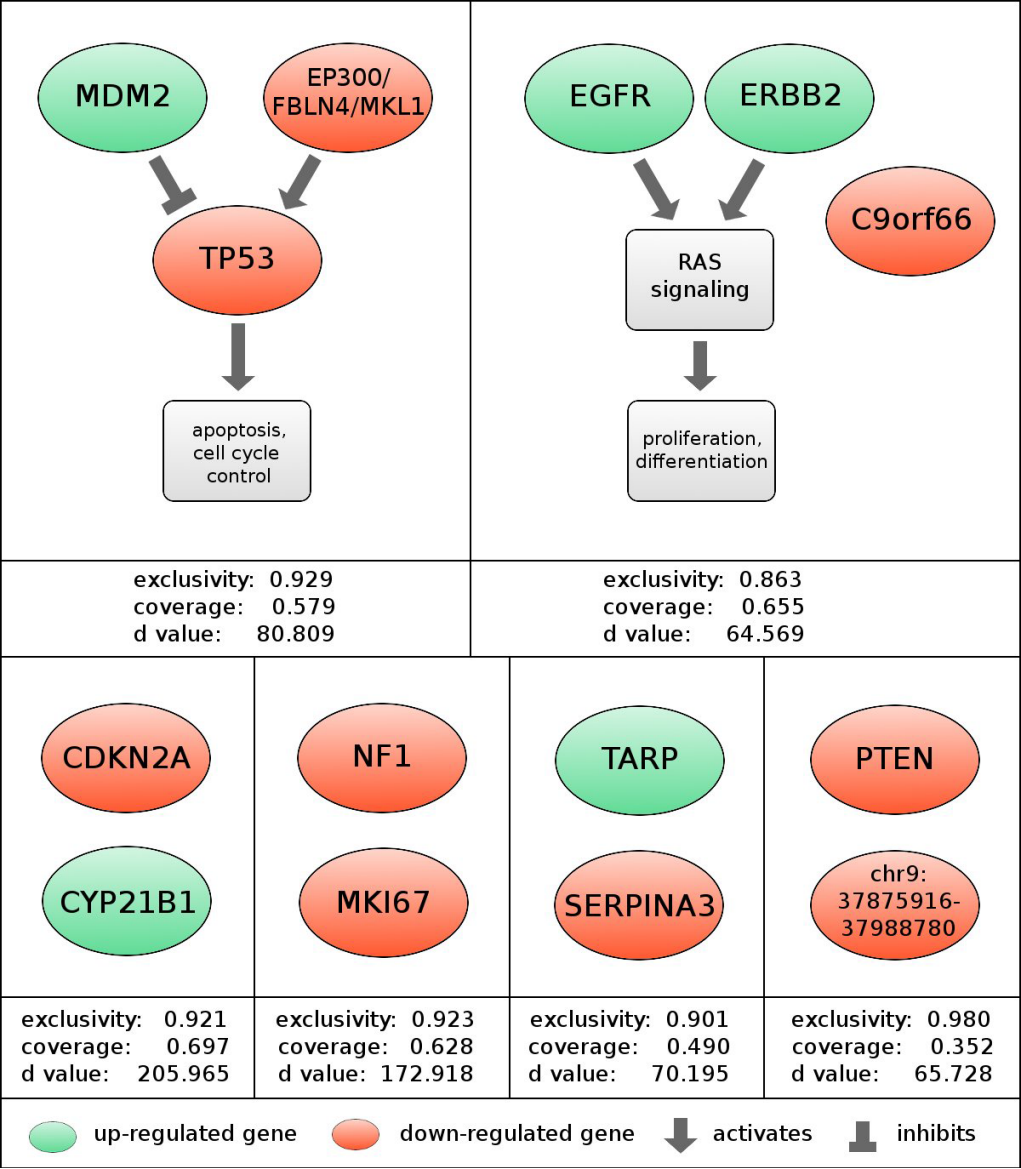
**Pathway context for RME modules found in glioblastoma**. Genes colored red are recurrently mutated in such a way that we expect loss of function, and those colored green are amplified or contain putatively activating mutations. The d-score is the algorithmic significance value, with significance being equal to 2^*-d*^. 1st row: Alterations in *MDM2*, *TP53*, and *EP300 *each result in less tumor suppression from *TP53*. *EGFR *and *ERBB2 *are both activators of the *RTK/RAS/PI3K *oncogenic signaling pathways. 2nd row: four new modules of size two that do not directly correspond to a known pathway.

We next applied this method to data from genomic assays run on 145 primary GBM tumor samples, using a conservative recurrence threshold of 10%. The modules were ranked by their algorithmic significance scores. The top six modules listed in Figure 4 exceeded a significance cutoff of 2^-50 ^(~= 8.88 × 10^-16^). Three of the six modules contain components of core GBM pathways reported by the TCGA consortium [1], which examined a subset of the 145 tumors we analyzed. After identification of modules was complete, we used a combination of automated annotation and manual examination to identify the common functional roles that module members may play.

### Rediscovery and expansion of known modules

The highest-scoring rediscovered module consisted of alterations to the genes *TP53 *and *MDM2 *along with a ~5 Mb recurrently deleted region on chromosome 22 (Figure 4). *TP53 *regulates a variety of oncogenic processes and mutations of *TP53 *have been reported in most tumor types [26]. *MDM2 *inhibits *TP53*-mediated transcription, and aberrations in the pair have often been reported as mutually exclusive [10]. The associated region on chr22 contains three potential driver genes. Of the three, *EP300 *seems to be the most likely driver, as its product is known to complex with and acetylate the product of *TP53*, and its disruption leads to decreased function of the *TP53 *product [27]. The other two genes are not known to interact with the *TP53 *pathway.

We expect that alterations in any of these three module components would disrupt the tumor-suppressive activity of *TP53*. Though the relationship of *MDM2 *and *TP53 *was central to one of the GBM pathways reported by the TCGA Consortium, the link between *EP300 *dysregulation and the *TP53 *pathway has not been previously reported in glioblastoma.

We also observed that the pattern of regulation in this module is concordant with our knowledge about the tumor-suppressive activity of the *TP53 *pathway. Because the information about the nature (up or down) of the aberrations was not encoded into our matrix, this agreement with prior knowledge serves as an additional validation of our results (Figure 4). We observe similar concordance in other known pathways.

The second rediscovered module consists of the genes *ERBB2*, *EGFR*, and *C9orf66*. Both *ERBB2 *and *EGFR *are signal transduction proteins that induce the RTK/RAS signaling pathway, causing unconstrained proliferation, and they can interact directly to form a heterodimer [28]. Our algorithm extended this pathway by adding the largely unstudied *C9orf66 *which contains non-synonymous point mutations in 15 tumors. This suggests that the product of *C9orf66 *may play a tumor suppressive role in the context of the *RAS *pathway.

The first module that does not directly map to a known pathway consists of the genes *CDKN2A *and *CYP21B1*. Deletions and mutations of *CDKN2A*, like those observed in this cohort, are common in cancer. The other gene, *CYP21B1*, was called as the representative for a small region of recurrent amplification on chromosome 12. This region has previously been identified as a coordinately-regulated oncogenic cluster, both in glioblastoma and other tumor types [29]. Among this cluster of adjacent genes is *CDK4*, a cyclin-dependent kinase that is an inhibitor of the *RB1 *tumor suppressor and known target of *CDKN2A*'s p16 product. The interaction between *CDK4 *and *CDKN2A *is part of the third and final core pathway of GBM, as defined by the TCGA consortium. We suggest that this interaction may be responsible for the mutational pattern we observe.

### Newly Discovered Modules

In addition to finding RME modules that are components of known pathways, we discovered modules that are previously unreported. Several have intriguing functional similarities, such as the pro-apoptotic roles of both *SHB*, which may be driving the deletion of a small region on chromosome 9, and *PTEN*. The discovery of these modules provides intriguing hypotheses about the related roles of these genes in tumorigenesis. Comprehensive descriptions and annotation of each module can be found in Additional file 1.

### *EP300 *predicts survival for patients with glioblastoma

**Figure 5:**
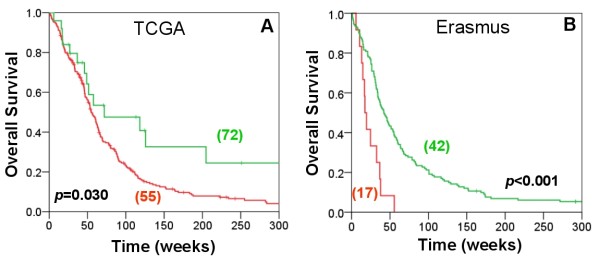
**EP300 expression predicts survival for patients with glioblastoma**. The role of *EP300 *in glioblastoma was validated by examining gene expression profiling data from two independent datasets of glioblastoma, TCGA (A) and Erasmus (B). Kaplan-Meier curves for 260 patients (A) and 153 patients (B) are shown. Samples were separated into high (green) and low (red) expression groups by recursive partitioning analysis. Median survival times for each group are shown in parentheses.

To show how one might leverage our gene module discovery process to produce clinically useful results, we decided to investigate EP300 further. Our method suggests that EP300 plays a role in the p53 pathway, which is strengthened by previous studies that show its interaction with *TP53*. Furthermore, *EP300 *aberrations have been observed in other types of cancer, but it has not been specifically linked to the progression of glioblastoma. Thus,we hypothesized that expression of *EP300 *may have value as a new prognostic indicator in glioblastoma. To validate this, we examined the relation between mRNA expression levels of *EP300 *and patient survival. Using two datasets, one from the TCGA and a second from the Netherlands (Erasmus), we examined the expression of *EP300 *in 260 and 153 cases, respectively. High *EP300 *expression was associated with improved survival (Figure 5), with a median survival of 72 weeks in the high-expressing cases vs. 55 weeks in the low-expressing for the TCGA cohort (p = 0.030, log-rank) and 42 weeks vs. 17 weeks for the Erasmus cohort (p < 0.001, log-rank). This survival prediction remained significant when adjusted for patient age, the most significant prognostic factor in glioblastoma [30] [p = 0.0141, HR = 1.81 (95 CI 1.12-3.18), for TCGA; p = 0.0112, HR = 1.55 (95 CI 1.12-2.08) for Erasmus, Cox proportional hazards model]. Interestingly, expression levels of TP53 and MDM2 do not have similar predictive value.

## Conclusions

We have developed a sensitive, simple, and fast method for automatically detecting functional modules in tumors based on patterns of recurrent genomic aberration alone. The results indicate that integrative analyses of genome characterization data have the potential to identify groups of genes that have related roles in producing cancer phenotypes. Furthermore, it is possible to generate hypotheses about pathway membership, or about the functional relevance of unexpected or uncharacterized genes by using co-occurrence in an RME module as an indicator of function.

Our experiments do show that RME patterns are not perfect. The fact that 30-70% of samples are not covered by individual modules may be explained by several factors, including low-frequency mutations that fall below our recurrence thresholds, the small proportion of genes that were assayed for somatic point mutations, and lack of comprehensive epigenomic assays, which could give information on gene silencing. As the costs of massively parallel sequencing drop, we expect more complete coverage of a larger number of samples, which may resolve the first two issues. A larger number of genes are also slated to be assayed for abnormal methylation patterns soon, and this algorithm can incorporate such data into future analyses. These comprehensive whole-genome data will undoubtedly improve our ability to detect functional modules and eliminate any bias that comes from operating on a reduced gene set.

We also note that while our method does not use pathway, interactome, and other network information, we do not suggest this method as a complete replacement for analyses that do use these data. In fact, we envision extensions of this method that may use background knowledge in a controlled and explicit way. At this point, we also do not make use of aberration co-occurrence, which may suggest a lack of functional similarity. Such overlapping aberrations do not lend themselves to the same kind of clear and compelling interpretation as RME patterns, but may be nonetheless useful in future expansions of this method.

As the throughputs of technologies and the capacity of data producing projects increases, so will the significance and abundance of RME patterns. In anticipation of this trend, this method has been designed at the outset to accommodate an increasing diversity and volume of genome characterization information. We therefore anticipate that the method will be increasingly useful in generating hypotheses that will drive specific experiments and increase understanding of cancer progression.

## Competing interests

The authors declare that they have no competing interests.

## Authors' contributions

CM and AM: project conception, algorithm design. CM: implementation, simulations, and application. SS, ES, KA: survival analysis. CM and AM manuscript preparation. AM project leadership and supervision. All authors read and approved the final manuscript.

## Pre-publication history

The pre-publication history for this paper can be accessed here:

http://www.biomedcentral.com/1755-8794/4/34/prepub

## Supplementary Material

Additional file 1 **Supplemental Methods and Results**. Click here for file

## References

[B1] Cancer Genome Atlas Consortium Comprehensive genomic characterization defines human glioblastoma genes and core pathways Nature 2008 455 1061 1068 10.1038/nature07385 18772890 PMC2671642

[B2] Eisen MB Spellman PT Brown PO Botstein D Cluster analysis and display of genome-wide expression patterns Proceedings of the National Academy of Sciences of the United States of America 1998 95 14863 14868 10.1073/pnas.95.25.14863 9843981 PMC24541

[B3] Basso K Margolin AA Stolovitzky G Klein U Dalla-Favera R Califano A Reverse engineering of regulatory networks in human B cells Nat Genet 2005 37 382 390 10.1038/ng1532 15778709

[B4] Segal E Friedman N Koller D Regev A A module map showing conditional activity of expression modules in cancer Nat Genet 2004 36 1090 1098 10.1038/ng1434 15448693 PMC2271138

[B5] Masayesva BG Ha P Garrett-Mayer E Pilkington T Mao R Pevsner J Speed T Benoit N Moon C Sidransky D Westra WH Califano J Gene expression alterations over large chromosomal regions in cancers include multiple genes unrelated to malignant progression Proceedings of the National Academy of Sciences of the United States of America 2004 101 8715 8720 10.1073/pnas.0400027101 15155901 PMC423261

[B6] Miklos GLG Maleszka R Microarray reality checks in the context of a complex disease Nat Biotechnol 2004 22 615 621 10.1038/nbt965 15122300

[B7] Tong AHY Lesage G Bader GD Ding H Xu H Xin X Young J Berriz GF Brost RL Chang M Chen Y Cheng X Chua G Friesen H Goldberg DS Haynes J Humphries C He G Hussein S Ke L Krogan N Li Z Levinson JN Lu H Menard P Munyana C Parsons AB Ryan O Tonikian R Roberts T Sdicu A Shapiro J Sheikh B Suter B Wong SL Zhang LV Zhu H Burd CG Munro S Sander C Rine J Greenblatt J Peter M Bretscher A Bell G Roth FP Brown GW Andrews B Bussey H Boone C Global Mapping of the Yeast Genetic Interaction Network Science 2004 303 808 813 10.1126/science.1091317 14764870

[B8] Gazdar AF Shigematsu H Herz J Minna JD Mutations and addiction to *EGFR*: the Achilles 'heal' of lung cancers? Trends Mol Med 2004 10 481 486 10.1016/j.molmed.2004.08.008 15464447

[B9] Tomlins SA Rhodes DR Perner S Dhanasekaran SM Mehra R Sun X Varambally S Cao X Tchinda J Kuefer R Lee C Montie JE Shah RB Pienta KJ Rubin MA Chinnaiyan AM Recurrent Fusion of TMPRSS2 and ETS Transcription Factor Genes in Prostate Cancer Science 2005 310 644 648 10.1126/science.1117679 16254181

[B10] Momand J Zambetti GP Olson DC George D Levine AJ The mdm-2 oncogene product forms a complex with the p53 protein and inhibits p53-mediated transactivation Cell 1992 69 1237 1245 10.1016/0092-8674(92)90644-R 1535557

[B11] Yeang C McCormick F Levine A Combinatorial patterns of somatic gene mutations in cancer FASEB J 2008 22 2605 2622 10.1096/fj.08-108985 18434431

[B12] Ross SM Introduction to Probability Models, Seventh Edition 2000 7 Academic Press

[B13] Olshen AB Venkatraman ES Lucito R Wigler M Circular binary segmentation for the analysis of array-based DNA copy number data Biostatistics 2004 5 557 572 10.1093/biostatistics/kxh008 15475419

[B14] Taylor BS Barretina J Socci ND DeCarolis P Ladanyi M Meyerson M Singer S Sander C Functional Copy-Number Alterations in Cancer PLoS ONE 2008 3 e3179 10.1371/journal.pone.0003179 18784837 PMC2527508

[B15] Littlestone N Learning Quickly When Irrelevant Attributes Abound: A New Linear-Threshold Algorithm Mach. Learn 1988 2 285 318

[B16] Milosavljevic A Jurka J Discovering simple DNA sequences by the algorithmic significance method Comput Appl Biosci 1993 9 4 407 411 8402207 10.1093/bioinformatics/9.4.407

[B17] Karplus K Using markov models and hidden markov models to find repetitive extragenic palindromic sequences in Escherichia Coli Technical Report UCSC-CRL-94-24 1994

[B18] Mitrophanov AY Borodovsky M Statistical significance in biological sequence analysis Brief Bioinform 2006 7 2 24 10.1093/bib/bbk001 16761361

[B19] Eddy SR A Probabilistic Model of Local Sequence Alignment That Simplifies Statistical Significance Estimation PLoS Comput Biol 2008 4 e1000069 10.1371/journal.pcbi.1000069 18516236 PMC2396288

[B20] Giancarlo R Scaturro D Utro F Textual data compression in computational biology: a synopsis Bioinformatics 2009 25 1575 1586 10.1093/bioinformatics/btp117 19251772

[B21] Milosavljevic A Discovering Dependencies via Algorithmic Mutual Information: A Case Study in DNA Sequence Comparisons Mach. Learn 1995 21 35 50

[B22] Gravendeel LA Kouwenhoven MC Gevaert O de Rooi JJ Stubbs AP Duijm JE Daemen A Bleeker FE Bralten LB Kloosterhof NK De Moor B Eilers PH van der Spek PJ Kros JM Sillevis Smitt PA van den Bent MJ French PJ Intrinsic Gene Expression Profiles of Gliomas Are a Better Predictor of Survival than Histology Cancer Res 2009 69 9065 9072 10.1158/0008-5472.CAN-09-2307 19920198

[B23] Dai M Wang P Boyd AD Kostov G Athey B Jones EG Bunney WE Myers RM Speed TP Akil H Watson SJ Meng F Evolving gene/transcript definitions significantly alter the interpretation of GeneChip data Nucleic Acids Res 2005 33 e175 e175 10.1093/nar/gni179 16284200 PMC1283542

[B24] Kaplan EL Meier P Nonparametric estimation from incomplete observations J.of the American Statistical Association 1958 53 457 48 10.2307/2281868

[B25] Cox DR Regression models and life tables J Royal Stat Soc 1972 34 187 220

[B26] Levine AJ p53, the cellular gatekeeper for growth and division Cell 1997 88 323 331 10.1016/S0092-8674(00)81871-1 9039259

[B27] Dornan D Shimizu H Perkins ND Hupp TR DNA-dependent acetylation of p53 by the transcription coactivator p300 J. Biol. Chem 2003 278 13431 13441 10.1074/jbc.M211460200 12499368

[B28] Goldman R Levy RB Peles E Yarden Y Heterodimerization of the erbB-1 and erbB-2 receptors in human breast carcinoma cells: a mechanism for receptor transregulation Biochemistry 1990 29 11024 11028 10.1021/bi00502a002 1980216

[B29] Kim H Huang W Jiang X Pennicooke B Park PJ Johnson MD Integrative genome analysis reveals an oncomir/oncogene cluster regulating glioblastoma survivorship Proceedings of the National Academy of Sciences 2010 107 2183 2188 10.1073/pnas.0909896107 PMC2836668 20080666

[B30] Sulman EP Guerrero M Aldape K Beyond Grade: Molecular Pathology of Malignant Gliomas Seminars in Radiation Oncology 2009 19 142 149 10.1016/j.semradonc.2009.02.001 19464628

