# Supplementary material for: Discovering functional modules by identifying recurrent and mutually exclusive mutational patterns in tumors
Source: BMC Med Genomics. 2011 Apr 14;4:34. doi: 10.1186/1755-8794-4-34 (PMC3102606; doi:10.1186/1755-8794-4-34)
Supplement: Additional file 1 — Supplemental Methods and Results. [file 1755-8794-4-34-S1.DOC]

Supplementary Data

**New module annotation:**

***MKI67* and *NF1***

The first new module consists of deletions and point mutations in the genes *MKI67* and *NF1,* covering 62% of tumors with 92% exclusivity. *NF1* has been previously identified as a tumor-suppressor gene in glioblastoma and functions by negatively regulating the *RAS* pathway and retarding cell growth (Cichowski et al 2003). Despite having an unknown function, *MKI67* has been used for decades as a measure of cell cycle progression and tumor growth rate. In quiescent cells, *MKI67* is almost entirely absent, but as a cell enters G1, *MKI67* becomes abundant and localizes to the nucleus, clustering around the centrosomes. Knockdown studies have shown that it is required for cell proliferation, and along with the FHA and DNA binding domains it contains, may suggest that it is involved in a cell-cycle checkpoint role, either recruiting DNA repair proteins if lesions have occurred, or allowing progression through mitosis (Endl and Gerdes 2000). If this is the case, inactivating mutations of *MKI67* may allow the cell to avoid the usual cell-cycle controls and enter a stage of unchecked growth. Further study is needed to elucidate how alterations in these two proteins might play complementary roles, but the hypothesis that inactivation of either may induce proliferation is intriguing.

***TARP* and *SERPINA3***

The genes *TARP* and *SERPINA3* comprise another module, which spans 71 tumors with 7 overlaps. *TARP* is present in both breast and prostate cancer cells and has been linked to increased growth rate and altered gene expression in prostate cancer cells (Wolfgang et al. 2001). *SERPINA3* is a serine protease with point mutations in 5 samples, and is part of a large deletion in 12 samples. It is presently unclear how they may fill a common functional niche.

***PTEN* and chr9 region**

The final module pairs the tumor suppressor gene *PTEN* with a small region of deletion on chromosome 9. Although only seen in 38% of tumors, it has 98% exclusivity. The deletion region contains two genes, *MCART1* and *SHB*. Though *MCART1* has no obvious connection to cancer, expression of *SHB* is essential for triggering apoptosis through endostatin-induced pathways (Dixelius et al. 2000). *PTEN* plays an analogous role by inducing apoptosis through the *PI3K/AKT* pathways (Weng et al. 2001), and this common role may explain the makeup of this functional module.

**Existing module annotation:**

***TP53*, *MDM2*, and *EP300/FBLN4/MKL1***

This module appears in 84 tumors, with just 6 of them containing more than one alteration. The most frequent alteration is point mutation of *TP53*, which occurs in 50 samples. Its inhibitor *MDM2* is amplified in another 17, and the EP300/FBLN4/MKL1 region is deleted in 22.

***ERBB2*, *EGFR*, and *C9orf66***

This module is altered in 80 tumors, with 13 overlapping alterations. Copy number amplification of *EGFR* occurs in 78% of the tumors, and these are complemented by a smaller number of base-pair changes in *EGFR* and *ERBB2*, which may correspond to activating mutations reported in other studies (Shigematsu 2005; Lynch et al 2004). Co-occurrence of *EGFR* amplification and mutation is also common, occurring 20 times. *C9orf66*, the third member of this module, has non-synonymous point mutations in 15 tumors

***CDKN2A* and *CYP21B1***

This module spans 111 samples, with just 8 containing more than one mutation. *CDKN2A* is just deleted in 77 tumors, has just point mutations in 1 tumor, and has both in another 3 tumors. The *CYP21B1* region is amplified in 25 tumors, with another 3 containing point mutations.

**Driver Gene selection and copy-number variant removal:**

Data from Agilent 244A copy number arrays were fed into the RAE algorithm (Taylor, 2008), using a permutation count of 100 and a qvalue threshold of 0.01. All focal peaks in the output was retained, and each broad peak was removed unless it did not contain a focal peak. To remove copy number variants, the Database of Genomic Variants build hg18.v7.mar.2009 was filtered to remove variants with the Method/Platform field matching any of: BAC Array CGH, FISH, Genotyping/PFGE, Mendelian inconsistencies, Null genotypes (Iafrate et al, 2004). This left only the high-resolution subset of the data, which was merged across data sets and any regions confirmed at least 5 times were kept. These CNVs were then compared to CN-altered peak regions, and any peak at least 50% covered by a CNV was removed

Putative driver genes for each remaining copy-number peak were selected by choosing the gene in a given peak with the highest number of validated somatic mutations. If no genes were present in the peak, miRNAs were used, and if neither was present, the peak was labeled with chromosome and position. If a driver gene was called, the peak size was reduced to the size of the gene, plus 10kb in either direction, to account for proximal regulatory regions. If a peak contained several genes with the same number of somatic mutations, the names were concatenated together, and peak size was reduced to include just the region containing the potential drivers. The resulting peaks were then intersected with segments of gain and loss from each sample to determine which samples contained the specified alteration.

**Winnow edge weight calculation**

In order to assess whether Winnow was better at eliminating false edges, we created 100 networks in the same manner described in the simulation section and seeded each with a single network of 50-100% coverage and 95% exclusivity. For each of these networks, we calculated pair-wise scores using both winnow and simple exclusivity scores, then determined the maximum threshold that could be used without removing the edges necessary to find the seeded module. Then, the number of edges exceeding this threshold were counted. As shown in Supplemental Figure 1, winnow allows us to filter more aggressively and create smaller, less connected networks. This becomes especially important as the sample sizes and the number of genes assayed increases, which is where winnow is most effective.


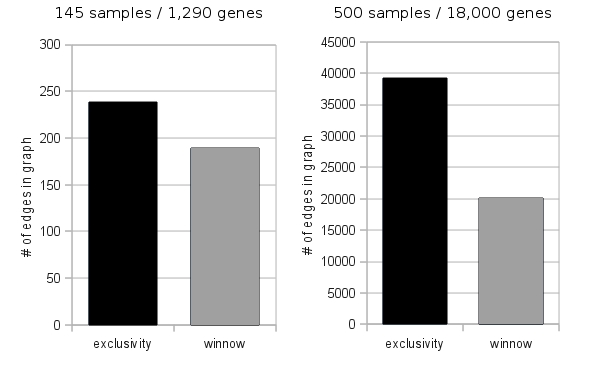


**Supplementary Figure 1:** Comparison of graphs generated using winnow vs. simple exclusivity-based methods

**Background Mutation Rate**

For copy number alterations, we utilize the McCarroll Hap-map data set (McCarroll et al, 2008), obtained from the Database of Genomic Variants (Iafrate et al, 2004( giving an estimate of 0.981% of genes copy-number altered per sample. For point-mutations, we use the estimate given in the Parsons paper (Parsons et al 2008) of 0.38 mutations per Mb of sequence. Combined, when considering the 1290 genes sequenced, this gives an expected passenger mutation rate of 13.38 genes altered per sample.

**Threshold Value**

The choice of significance threshold value was informed by several iterations of testing on simulated data, before ultimately choosing a value of 2^-50^ that produces strong sensitivity and precision for the input matrix size.

**Sorting the mutation matrix**

The RME Algorithm first sorts the samples, then the genes, by their sums, placing the most frequently altered samples at the top of the matrix, and the aberrations at the beginning of each row. Ties are resolved by a lexicographic sorting by sample and gene identifier respectively.

As a penalty for doing so, we reduce the score by the number of bits necessary to represent the new sorted order. According to combinatorial theory there would be *n*! possible permutations of rows, but the list of row sums may contain non-unique values. So if there are *r* rows with sum *s*, then the overall number of unique permutations for those rows is reduced to $n!/\coprod_{s}^{k} r_{s}!$, and the number of bits required to represent this order is obtained by taking the logarithm. For performance reasons, we compute the factorials in logarithmic space, and thus

Similarly, for sorting the columns, denotes the number of columns with sum *s*, and the penalty is:

These values are subtracted from the final value of d for each module.

**Comparison to other Methods**


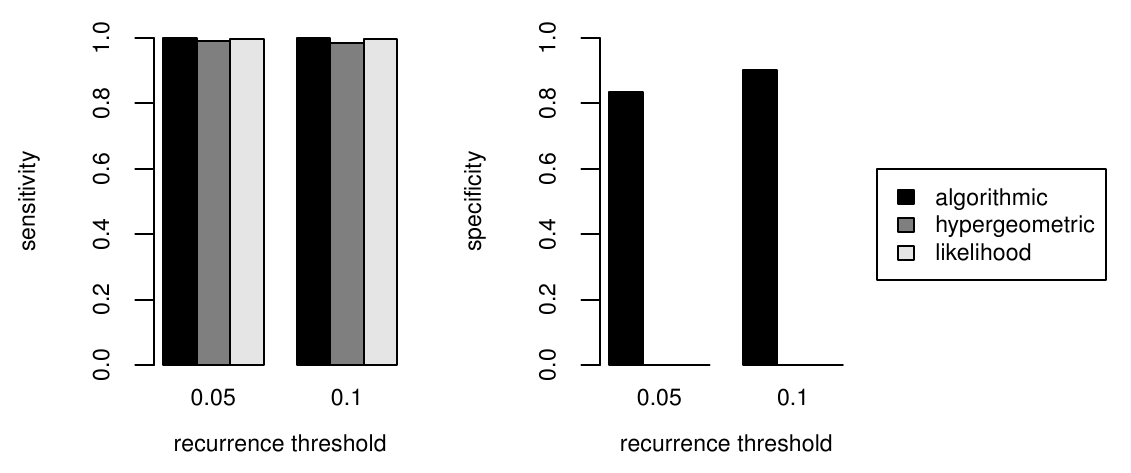


**Supplementary Figure 2:** When tested on large matrices seeded with RME patterns, algorithmic significance has greater precision than other methods designed for calculating the significance of mutually exclusive patterns.

**Automated Annotation**
In order to determine what the functional relevance of a module might be, automated annotation was performed using a in-house script that accesses the Database for Annotation, Visualization and Integrated Discovery (DAVID) (Dennis et al, 2003). We retrieve functional annotations including Gene Ontology terms, KEGG and BioCarta pathways, and protein domains, then filter and report only the annotations that are common to all members of the module. A full listing appears in the Supplemental Table 2. Another script was used to summarize the type and directionality (gain or loss) of each aberration.

References

Cichowski, K., Santiago, S., Jardim, M., Johnson, B.W., and Jacks, T. 2003. Dynamic

regulation of the Ras pathway via proteolysis of the NF1 tumor suppressor. Genes

& Development 17: 449-454.

Dennis, G., Sherman, B., Hosack, D., Yang, J., Gao, W., Lane, H.C., and Lempicki, R.

2003. DAVID: Database for Annotation, Visualization, and Integrated Discovery.

Genome Biology 4:P3.

Dixelius, J., Larsson, H., Sasaki, T., Holmqvist, K., Lu, L., Engstrom, A., Timpl, R.,

Welsh, M., and Claesson-Welsh, L. 2000. Endostatin-induced tyrosine kinase

signaling through the Shb adaptor protein regulates endothelial cell apoptosis.

Blood 95: 3403-3411.

Endl, E., and Gerdes, J. 2000. The Ki-67 Protein: Fascinating Forms and an Unknown

Function. Experimental Cell Research 257: 231-237.

Iafrate, A.J., Feuk, L., Rivera, M.N., Listewnik, M.L., Donahoe, P.K., Qi, Y., Scherer,

S.W., and Lee, C. 2004. Detection of large-scale variation in the human genome.

Nat Genet 36: 949-951.

Littlestone, N. Learning Quickly When Irrelevant Attributes Abound: A New Linear-

Threshold Algorithm. Mach. Learn. 2, 285-318 (1988).

Lynch, T.J. et al. Activating Mutations in the Epidermal Growth Factor Receptor

Underlying Responsiveness of Non-Small-Cell Lung Cancer to Gefitinib. N Engl J Med 350, 2129-2139 (2004).

McCarroll, S.A. et al. 2008. Integrated detection and population-genetic analysis of SNPs

and copy number variation. Nat Genet 40: 1166-1174.

Parsons, D.W. et al. 2008. An Integrated Genomic Analysis of Human Glioblastoma

Multiforme. Science 321: 1807-1812.

Shigematsu, H. et al. Somatic mutations of the HER2 kinase domain in lung

adenocarcinomas. Cancer Res 65, 1642-1646 (2005).

Taylor, B.S. et al. 2008. Functional Copy-Number Alterations in Cancer. PLoS ONE 3:

e3179.

Weng, L., Brown, J.L., and Eng, C. 2001. PTEN induces apoptosis and cell cycle arrest

through phosphoinositol-3-kinase/Akt-dependent and -independent pathways.

Hum. Mol. Genet. 10: 237-242.

Wolfgang, C.D., Essand, M., Lee, B., and Pastan, I. 2001. T-Cell Receptor {gamma} Chain

Alternate Reading Frame Protein (TARP) Expression in Prostate Cancer Cells Leads to

an Increased Growth Rate and Induction of Caveolins and Amphiregulin. Cancer Res 61: 8122-8126. 11.

Yeang C, McCormick F, Levine A: Combinatorial patterns of somatic gene mutations in cancer. FASEB J. 2008,

22:2605-2622

**Supplemental Table 1:** complete TCGA sample list

TCGA-02-0001-01C
TCGA-02-0003-01A
TCGA-02-0006-01B
TCGA-02-0007-01A
TCGA-02-0009-01A
TCGA-02-0010-01A
TCGA-02-0011-01B
TCGA-02-0014-01A
TCGA-02-0021-01A
TCGA-02-0024-01B
TCGA-02-0027-01A
TCGA-02-0028-01A
TCGA-02-0033-01A
TCGA-02-0034-01A
TCGA-02-0037-01A
TCGA-02-0038-01A
TCGA-02-0043-01A
TCGA-02-0046-01A
TCGA-02-0047-01A
TCGA-02-0052-01A
TCGA-02-0054-01A
TCGA-02-0055-01A
TCGA-02-0057-01A
TCGA-02-0058-01A
TCGA-02-0060-01A
TCGA-02-0064-01A
TCGA-02-0069-01A
TCGA-02-0071-01A
TCGA-02-0074-01A
TCGA-02-0075-01A
TCGA-02-0080-01A
TCGA-02-0083-01A
TCGA-02-0085-01A
TCGA-02-0086-01A
TCGA-02-0089-01A
TCGA-02-0099-01A
TCGA-02-0102-01A
TCGA-02-0107-01A
TCGA-02-0113-01A
TCGA-02-0114-01A
TCGA-02-0115-01A
TCGA-02-0116-01A
TCGA-06-0122-01A
TCGA-06-0124-01A
TCGA-06-0125-01A
TCGA-06-0126-01A
TCGA-06-0128-01A
TCGA-06-0129-01A
TCGA-06-0130-01A
TCGA-06-0133-01A
TCGA-06-0137-01A
TCGA-06-0139-01B
TCGA-06-0141-01A
TCGA-06-0142-01A
TCGA-06-0143-01A
TCGA-06-0145-01A
TCGA-06-0147-01A
TCGA-06-0148-01A
TCGA-06-0169-01A
TCGA-06-0138-01A
TCGA-06-0150-01A
TCGA-06-0151-01A
TCGA-06-0154-01A
TCGA-06-0156-01A
TCGA-06-0157-01A
TCGA-06-0158-01A
TCGA-06-0159-01A
TCGA-06-0160-01A
TCGA-06-0165-01A
TCGA-06-0166-01A
TCGA-06-0167-01A
TCGA-06-0168-01A
TCGA-06-0171-01A
TCGA-06-0173-01A
TCGA-06-0174-01A
TCGA-06-0176-01A
TCGA-06-0178-01A
TCGA-06-0184-01A
TCGA-06-0185-01A
TCGA-06-0187-01A
TCGA-06-0188-01A
TCGA-06-0189-01A
TCGA-06-0190-01A
TCGA-06-0195-01B
TCGA-06-0197-01A
TCGA-06-0201-01A
TCGA-06-0206-01A
TCGA-06-0208-01B
TCGA-06-0209-01A
TCGA-06-0210-01B
TCGA-06-0211-01B
TCGA-06-0213-01A
TCGA-06-0214-01A
TCGA-06-0216-01A
TCGA-06-0219-01A
TCGA-06-0221-01A
TCGA-06-0237-01A
TCGA-06-0241-01A
TCGA-02-0015-01A
TCGA-02-0016-01A
TCGA-02-0023-01B
TCGA-02-0025-01A
TCGA-02-0026-01B
TCGA-02-0048-01A
TCGA-02-0068-01A
TCGA-02-0070-01A
TCGA-02-0104-01A
TCGA-08-0244-01A
TCGA-08-0245-01A
TCGA-08-0246-01A
TCGA-08-0344-01A
TCGA-08-0347-01A
TCGA-08-0348-01A
TCGA-08-0350-01A
TCGA-08-0351-01A
TCGA-08-0353-01A
TCGA-08-0354-01A
TCGA-08-0355-01A
TCGA-08-0356-01A
TCGA-08-0357-01A
TCGA-08-0359-01A
TCGA-08-0360-01A
TCGA-08-0375-01A
TCGA-08-0380-01A
TCGA-08-0389-01A
TCGA-08-0390-01A
TCGA-02-0079-01A
TCGA-02-0084-01A
TCGA-06-0127-01A
TCGA-06-0152-01A
TCGA-06-0238-01A
TCGA-06-0644-01A
TCGA-06-0645-01A
TCGA-06-0646-01A
TCGA-06-0648-01A
TCGA-08-0345-01A
TCGA-08-0349-01A
TCGA-08-0352-01A
TCGA-08-0358-01A
TCGA-08-0373-01A
TCGA-08-0386-01A
TCGA-12-0616-01A
TCGA-12-0618-01A
TCGA-12-0619-01A
TCGA-12-0620-01A

**Supplemental Table 2**: Listing of common functional annotations for each module

| module | score | exclusivity | coverage | selected common GO terms | BIOCARTA pathways | KEGG pathways | notes |
| --- | --- | --- | --- | --- | --- | --- | --- |
| TP53, MDM2, EP300 | 80.8091 | 0.92857 | 0.5793 | regulation of gene expression, regulation of transcription,  transcription from RNA polymerase II promoter,  protein modification process,  RNA biosynthetic process | h_g2Pathway:Cell Cycle: G2/M Checkpoint, h_p53hypoxiaPathway:Hypoxia and p53 in the Cardiovascular system | hsa05215:Prostate cancer,  hsa04110:Cell cycle |  |
| ERBB2, EGFR,  (C9orf66) | 64.549 | 0.86316 | 0.655 | positive regulation of cell proliferation, regulation of kinase activity,  enzyme linked receptor protein signaling pathway,  cell surface receptor linked signal transduction, | h_her2Pathway:Role of ERBB2 in Signal Transduction and Oncology,  h_tffPathway:Trefoil Factors Initiate Mucosal Healing | hsa04012:ErbB signaling pathway,  hsa05215:Prostate cancer,  hsa05212:Pancreatic cancer,  hsa05213:Endometrial cancer,  hsa05223:Non-small cell lung cancer hsa04510:Focal adhesion | C9orf66 not present in database |
| MKI67, NF1 | 172.918 | 0.923 | 0.628 | regulation of progression through cell cycle, intracellular membrane-bound organelle | none | none |  |
| CDKN2A, CYP27B1 | 205.965 | 0.921 | 0.697 | intracellular membrane-bound organelle, DNA-dependent | none | none | CYP27B1 representative of oncogenic cluster -other members may have more commonality |
| TARP, SERPINA3 | 70.195 | 0.901 | 0.490 | n/a | n/a | n/a | TARP not found in database |
| PTEN, SHB | 65.728 | 0.980 | 0.352 | apoptosis, angiogenesis, organ morphogenesis, cell differentiation, signal transduction | none | none | using SHB as representative for chr9:37875916-37988780 region |
